# Determining causal pathogens and inflammatory state of mastitis in dairy cows via Gram staining of precipitates in milk

**DOI:** 10.3389/fvets.2024.1492564

**Published:** 2025-01-13

**Authors:** Naoki Suzuki, Naoki Isobe

**Affiliations:** Graduate School of Integrated Sciences for Life, Hiroshima University, Higashihiroshima, Hiroshima, Japan

**Keywords:** diagnosis, mastitis, Gram staining, bovine, inflammation

## Abstract

Early detection of bovine mastitis-causing pathogens is necessary for treatment. As culturing methods are time-consuming, a more rapid detection technique is required. This study investigated the sensitivity, specificity, and detection limit of Gram staining of milk precipitates (milk Gram stain, MGS) to detect bovine mastitis-causing pathogens in milk, as well as the potential of MGS to diagnose inflammation by counting polymorphonuclear leukocytes (PMN). MGS was performed on spontaneous mastitis cases. Culture methods were also used as reference standards to calculate the sensitivity, specificity, and bacterial load in milk to determine the detection limit of MGS. PMN in the mastitic milk were counted using Gram staining. Further, somatic cell counts (SCC), interleukin (IL)-6, IL-8, tumor necrosis factor (TNF)-α, IL-10 and serum amyloid A (SAA) concentrations in mastitic milk were measured using cell counting and enzyme-linked immunosorbent assay. The sensitivity and specificity for all pathogens were 0.62 and 0.90, for Gram-positive were 0.67 and 0.90, and for Gram-negative were 0.50 and 1.00, respectively. The detection limits for Gram-positive and Gram-negative bacteria were 1,560 and 4,680 cfu/mL, respectively. SCC were significantly positively correlated with PMN, milk IL-6, TNF-α, and SAA concentrations, whereas PMN were significantly negatively correlated with milk IL-10 concentration. Our results suggest that MGS may be applied as a rapid method to identify causal pathogens of mastitis before culture results are determined and may also estimate inflammatory status which cannot be detected with SCC. Further clinical trials are required to elucidate whether MGS is useful in clinical veterinary settings.

## 1 Introduction

Mastitis in dairy cows is caused by intramammary infection (IMI) which results in inflammation of the mammary glands. In the dairy industry, mastitis causes reduced milk production, disrupting the stable supply of milk, thereby causing large economic losses to dairy farmers (1, 2). In addition, because several causal pathogens of IMI are zoonotic, IMI has food safety implications in dairy products (3). A wide variety of pathogens can cause IMI, including bacteria, fungi, and algae (4–6).

Diagnosing the causal pathogens of IMI is important to develop treatment strategies for mastitis in dairy cows. The use of antimicrobial agents for treating mastitis caused by non-bacterial pathogens, such as fungi or algae, has no therapeutic effect. In addition, the dispensable use of antimicrobials in food-producing animals may cause the transmission of antimicrobial resistant bacteria or their related genes to the public (7). Recent studies suggest that intramammary infusion of 3rd generation cephalosporins for non-severe mastitis caused by Gram-negative pathogens, such as *Escherichia coli* or *Klebsiella pneumoniae*, does not improve production losses and milk somatic cell counts, which are indicators of mammary gland inflammation (8). However, Tomazi et al. suggested that antimicrobial use remains an indispensable strategy for treating clinical mastitis caused by Gram-positive bacteria (9). Although treatment strategies, particularly antimicrobial use, should be developed after identifying the causal pathogens, the recommendation to use culturing to identify pathogens forces veterinarians to treat empirically until the culture results are obtained.

To rapidly detect the causal pathogens of mastitis, culture-independent methods, such as PCR, loop-mediated isothermal amplification (10) and fluorescent *in situ* hybridization (11), have been developed. We have also reported the Gram staining of milk precipitates obtained via centrifugation (milk Gram stain, MGS) to improve the background of microscopic images; the sensitivity and specificity of MGS for detecting Gram-positive bacteria were 0.84 and 0.86, and for Gram-negative were 0.50 and 0.95, respectively (4). Rapid diagnosis of causal pathogens of bacterial infection via Gram staining has been applied in human hospitals (12). This simple and inexpensive method is easy to implement in veterinary settings, where diagnostic equipment is limited, but prudent use of antimicrobial agents is required. However, to the best of our knowledge, the accuracy of MGS in detecting causal pathogens of mastitis has not been reported, except in our previous study (4). Thus, veterinarians cannot obtain the information necessary for its clinical application, such as its reproducibility and detection limits.

We also detected polymorphonuclear leukocytes (PMN) in the MGS (4). Milk somatic cells are mostly leukocytes with few mammary epithelial cells (13). PMN counts in the local secretory fluid have been investigated as an indicator of infectious diseases, such as urinary infection (14). Odeh suggested that the tumor necrosis factor (TNF)-α level was positively correlated with PMN counts in the pleural fluid of patients with complicated parapneumonic effusion (15). In veterinary settings, endometrial PMN count has been used as an indicator of clinical and subclinical endometritis in dairy cows (16). Despite the recent suggestion that PMN counts or the differential cell count in milk, which is the percentage of PMN combined with lymphocytes, could be a new parameter for mastitis screening (17, 18), the relationship between PMN counts in milk and mammary gland inflammation remains unclear.

This study aimed to investigate the accuracy and detection limit of MGS to detect causal pathogens of mastitis. In addition, we investigated the potential of MGS to diagnose inflammation by counting PMN in the same visual field of detecting pathogens.

## 2 Materials and methods

### 2.1 Study design

Mastitic milk samples from Holstein-Friesian cows were collected in Hiroshima Prefecture and sent to Hiroshima University to calculate the sensitivity, specificity, and detection limit of MGS. This study was conducted in accordance with the guidelines for animal experiments issued by Hiroshima University and was approved by the Animal Research Committee of Hiroshima University (E19-3).

### 2.2 Definitions of cases, collecting milk samples, and measuring somatic cell counts (SCC)

All mastitis cases enrolled in this study were spontaneous and occurred on commercial dairy farms. Mastitis was classified as mild, moderate, or severe (19). Mild cases showed only abnormal milk (abnormal color, viscosity, or consistency), moderate cases showed abnormal milk and affected quarters (heat, pain, redness, or swelling of the quarter), and severe cases showed systemic symptoms (depression, anorexia, dehydration, or fever) in addition to milk and quarter symptoms. Quarter-milk samples from mastitic udders were collected aseptically. The teats were debrided with 70% alcohol swabs, and foremilk of three or more hand-squeezed milk samples was discarded. Approximately 10 mL of milk were collected into a sterilized tube and immediately stored at 4°C until microbiological examinations and MGS. Further, 1 mL aliquot of milk was divided to measure SCC and stock skimmed milk samples. SCC was measured using a cell counter (Cell Counter DCC, DeLaval, Tumba, Sweden) following the recommended procedure.

### 2.3 MGS

MGS was conducted according to a previously described protocol with modifications (4). Briefly, 10 mL of milk were centrifuged at 500 × *g* for 5 min at 4°C. Subsequently, the milk fat and skimmed milk layers were removed. The remaining cell pellet was resuspended in 1 mL of sterilized saline. The cell pellet was concentrated 10 times, smeared on a glass slide, fixed with 99.8% methanol for 2 min, and Gram stained (0.2% Victoria blue solution for 1 min, 2% picric acid solution with methanol for 1 min, and 0.25% safranin solution for 1 min; Favor G, Nissui Pharmaceutical Co., Ltd., Tokyo, Japan). The 2 mL of removed skimmed milk were stored at −20°C until enzyme-linked immunosorbent assay (ELISA).

### 2.4 Bacterial culturing as the reference standard and quantification of bacterial loads

Milk culture is considered the gold standard for detecting causal pathogens of mastitis (10); the National Mastitis Council also recommends culture-based methods for the etiological diagnosis of mastitis. We used culture-based methods and modified them as reference standards for the purpose of this study. Milk samples were diluted with sterilized phosphate-buffered saline at 10, 100 and 1,000 times to calculate colony forming units (cfu), and 100 μL each were inoculated onto chromogenic agar plate (CHROMagar™ Orientation, Kanto Chemical Co., Inc., Tokyo, Japan). Only undiluted milk samples were inoculated onto blood agar plate (trypticase soy agar with 5% sheep blood; AccuRate™ Sheep Blood Agar, Shimadzu Diagnostics Corporation, Tokyo, Japan) to detect nutrient-demanding bacteria. Plates were incubated at 37°C for 24–48 h. Colony counts were conducted for each colony with different morphologies on chromogenic agar plate. Gram staining was performed for all isolates on chromogenic agar plates; if the number of colonies on the blood agar medium exceeded those on the chromogenic agar plate, Gram staining was performed with the colonies on the blood agar plates. Milk samples with three or more colony types were considered contaminated (20). All isolates were pure-cultured and stored at −80°C until identification.

### 2.5 16S rRNA gene sequencing for identification of isolates

All isolates were subjected to 16S rRNA gene sequencing to identify bacterial species. Bacterial DNA was extracted using the Fungal/Bacterial Miniprep Kit and Quick-DNA (Zymo Research, Carlsbad, CA, USA), according to the manufacturer's instructions. The fragments of bacterial 16S rRNA gene were amplified via PCR with primers 10F (5′-GTTTGATCCTGGCTCA-3′) and 800R (5′-TACCAGGGTATCTAATCC-3′). The 16S rRNA gene was amplified with the following cycle conditions: 94°C for 3 min, followed by 30 cycles of 94°C for 30 s, annealing at 51°C for 60 s, elongation at 72°C for 60 s, and final elongation at 72°C for 7 min. All amplification products were visualized with electrophoresis on 1.0% agarose gel and fluorescent dye. Amplification products were purified for gene sequencing using a PCR Product clean-up reagent kit (ExoSAP-IT^TM^; Thermo Fisher Scientific, Waltham, MA, USA), and sequencing was performed using an Applied Biosystem 3500xL Genetic Analyzer (Thermo Fisher Scientific). The bacterial species of the isolates were identified using the BLAST algorithm from the National Center for Biotechnology Information (https://blast.ncbi.nlm.nih.gov/Blast.cgi). Sequences with >98% similarity were considered to belong to the same species. After identification of the isolates, the sensitivity and specificity of the MGS for the detection of pathogens were calculated. Achievement of bacterial detection was defined as the same bacterial color (gram-positive or gram-negative), and morphology was observed in both MGS and the identified pathogens (4).

### 2.6 PMN counts

PMN were counted under a microscope with a randomly selected visual field. Cells were counted until 100 counts or 10 visual fields were obtained. The percentage of PMN in the somatic cells was calculated using the following formula:


$$\begin{array}{l} PMN\;\left(\%\right)=\frac{number\;of\;PMN}{number\;of\;somatic\;cells} \end{array}$$

To validate the accuracy of PMN counting using Gram staining, 30 cell suspensions from 30 mastitis cases were stained using both Giemsa and Gram staining, and the PMN counts were compared.

### 2.7 ELISA

The milk interleukin (IL)-6, tumor necrosis factor (TNF)-α, and serum amyloid A (SAA) concentrations were measured as the proinflammatory indicator; the milk IL-8 concentration was measured as the strength of neutrophil migration; and the milk IL-10 concentration was measured as the anti-inflammatory indicator. IL-6, IL-8, TNF-α, IL-10, and SAA concentrations were measured using an ELISA commercial kit (IL-6, IL-8, TNF-α, and IL-10; Cloud-Clone Corp., Hubei, China; and SAA; Immunology Consultants Laboratory, Inc., Tigard, OR, USA), according to the manufacturer's instructions.

### 2.8 Statistical analysis and data visualization

All statistical analyses were performed using the JMP Pro 16 software (SAS Institute Inc., Cary, NC, USA). Non-parametric methods were applied to the bacterial load (cfu/mL), and the Kruskal–Wallis test was performed to compare the bacterial load between bacterial characteristics and symptoms of mastitis (mild, moderate, or severe). Bacterial loads were logarithmically transformed to visualize the data. PMN (%) were normally distributed, whereas SCC, IL-6, IL-8, TNF-α, IL-10, and SAA concentrations in milk were lognormally distributed and logarithmically transformed to apply parametric methods. Pearson correlation coefficient was calculated between PMN (%) by Giemsa and Gram staining, and among SCC, PMN (%), IL-6, IL-8, TNF-α, IL-10, and SAA concentrations in milk. To calculate the detection limit of the MGS, ROC curve analysis was performed to define the cut-off. Statistical significance was set at *p* < 0.05.

## 3 Results

A total of 196 cases of mastitis were enrolled in this study. Of these, 52 milk samples were excluded because of low amounts of milk collected or gross contamination with foreign substances. Consequently, 144 mastitic milk samples from 144 cases were used in this study. Of these, 63, 12, and 5 cases were classified as mild, moderate, and severe, respectively. The remaining 64 samples could not be classified owing to missing records.

### 3.1 Bacteriological examination

Among the 144 mastitis cases, 49 were caused by one bacterial species, 20 were caused by two bacterial species, and no isolated bacteria were observed on all agars in 48 cases. The remaining 27 cases were classified as contaminated. The numbers of isolates are shown in Table 1. Thirty strains could not be identified with 10F-800R rRNA partial gene sequencing. Among the Gram-positive bacteria, 58 Gram-positive cocci, including *Staphylococcus, Streptococcus, Enterococcus*, and *Aerococcus* spp., and 6 Gram-positive bacilli, including *Bacillus, Paenibacillus*, and *Rothia* spp., were isolated. Among the Gram-negative bacteria, 11 *Enterobacteriales*, including *Escherichia, Klebsiella*, and *Enterobacter* spp., and 17 other non-fermenting Gram-negative bacteria (NF-GNB), including *Acinetobacter, Elizabethkingia, Pseudomonas, Chryseobacterium, Stenotrophomonas*, and *Sphingobacterium* spp., were isolated.

**Table 1:** Bacterial species isolated from mastitic milk.

**Bacterial species**	**Number**
**Gram-positives**	
**Gram-positive cocci**	
*Staphylococcus aureus*	5
*Staphylococcus simulans*	5
*Staphylococcus epidermidis*	4
*Staphylococcus chromogenes*	4
*Staphylococcus haemolyticus*	2
*Staphylococcus condimenti*	1
Staphylococci (unidentified)	12
*Streptococcus uberis*	5
*Streptococcus dysgalactiae* subsp. *dysgalactiae*	3
*Streptococcus equinus*	3
Streptococci (unidentified)	7
*Enterococcus saccharolyticus* subsp. *saccharolyticus*	3
*Enterococcus faecalis*	2
*Enterococcus faecium*	1
*Aerococcus* (unidentified)	1
**Gram-positive bacilli**	
*Bacillus australimaris*	2
*Bacillus licheniformis*	1
*Bacillus* (unidentified)	1
*Paenibacillus* (unidentified)	1
*Rothia terrae*	1
**Gram-negatives**	
***Enterobacteriales***	
*Escherichia coli*	5
*Klebsiella pneumoniae*	5
*Enterobacter* (unidentified)	1
**Other gram-negatives (non-fermenting)**	
*Acinetobacter* (unidentified)	7
*Elizabethkingia anophelis*	4
*Pseudomonas aeruginosa*	2
*Chryseobacterium vaccae*	2
*Stenotrophomonas maltophilia*	1
*Sphingobacterium* (unidentified)	1
Total isolates	92

### 3.2 Sensitivity and specificity of MGS

The sensitivity and specificity of the MGS are presented in Table 2. The sensitivity and specificity for all pathogens were 0.62 and 0.90, for Gram-positive were 0.67 and 0.90, and for Gram-negative were 0.50 and 1.00, respectively. Among the Gram-positive bacteria, the sensitivities of *Staphylococcus* sp. (*n* = 33) and *Streptococcus* sp. (*n* = 18) were 0.66 and 0.78, respectively. Among the Gram-negative bacteria, the sensitivities of *Enterobacteriales* and NF-GNB were 0.18 and 0.71, respectively. When symptoms were divided into mild (75 cases) or moderate and severe (17 cases), mild cases showed a sensitivity and specificity of 0.74 and 0.90, and moderate/severe cases showed 0.67 and 0.50, respectively.

**Table 2:** Sensitivity and specificity of MGS and bacterial load in each type of bacteria.

**Classification**	**Sensitivity**	**Specificity**	**Bacterial load (cfu/mL)**	
			**Mean**	**Median**
**Pathogen**				
All	0.62	0.90	155,703	760
Gram-positives	0.67	0.90	192,502	840
Staphylococcus	0.66	NC	284,533	1,560
Streptococcus	0.78	NC	77,422	1,300
Gram-negatives	0.50	1.00	63,706	560
*Enterobacteriales*	0.18	NC	4,647	440
NF-GNB	0.71	NC	107,016	800
**Severity**				
Mild	0.74	0.90	226,055	780
Moderate/severe	0.67	0.50	136,031	560

NF-GNB, non-fermenting Gram-negative bacteria; NC, not calculated.

### 3.3 Bacterial load and detection limit of MGS

**Figure 1:**
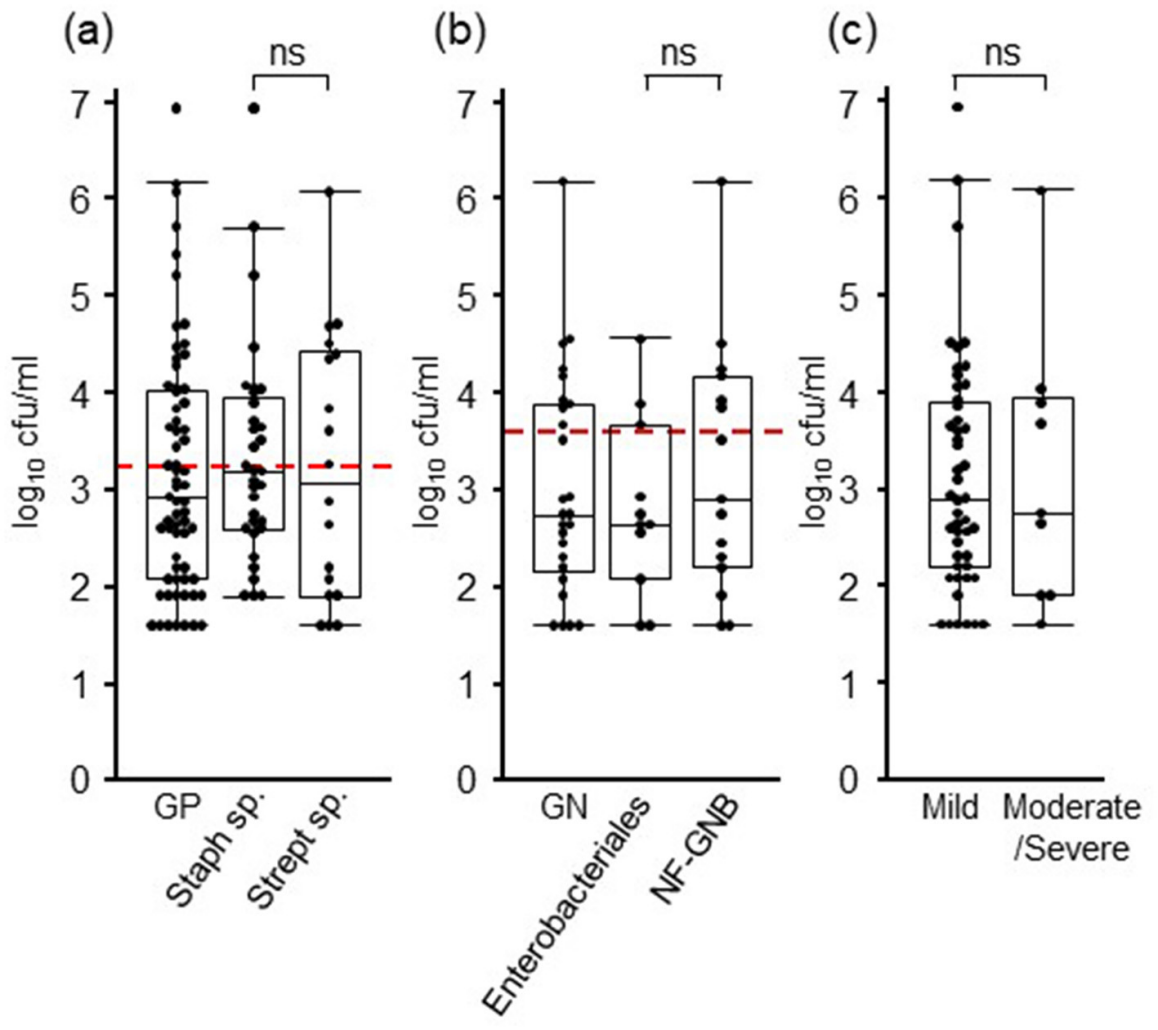
Bacterial loads in milk of mastitis cases. **(A)** The results of Gram-positive (GP) bacteria are shown, and those of *Staphylococcus* and *Streptococcus* species are considered the major Gram-positive pathogens of mastitis. **(B)** The results of Gram-negative (GN) bacteria are shown, and those of *Enterobacteriales* and non-fermenting gram-negative bacteria (NF-GNB) are considered the major Gram-negative pathogens of mastitis. **(C)** The results of 63 mild and 17 moderate/severe cases are presented. Red dotted lines indicate the detection limit calculated with ROC curve (1,560 and 4,680 cfu/mL for GN and GP, respectively). Box plots show lowest, first, second, third, and highest number from bottom to top except for outliers, respectively. cfu, colony-forming units; ns, no significant differences.

The bacterial loads of each isolate are listed in Table 2. In Gram-positive bacteria, the mean and median values of bacterial load were 192,502 and 840 cfu/mL, those of *Staphylococcus* sp. were 284,533 and 1,560 cfu/mL, and those of *Streptococcus* sp. were 77,422 and 1,300 cfu/mL, respectively. In Gram-negative bacteria, the mean and median values of bacterial load were 63,706 and 560 cfu/mL, those of *Enterobacteriales* were 4,647 and 440, and those of NF-GNB were 107,016 and 800 cfu/mL, respectively. There were no significant differences in the bacterial load between Gram-positive and Gram-negative bacteria, *Staphylococcus* and *Streptococcus*, and *Enterobacteriales* and NF-GNB (Figures 1A, B). The detection limits for Gram-positive bacteria, Gram-negative bacteria, and all pathogens calculated from the ROC analysis were 1,560, 4,680, and 1,560 cfu/mL, respectively. When comparing the bacterial load based on severity, no significant differences were observed between the mild and moderate/severe groups (Figure 1C).

### 3.4 PMN counts and concentrations of cytokines and SAA in milk

**Figure 2:**
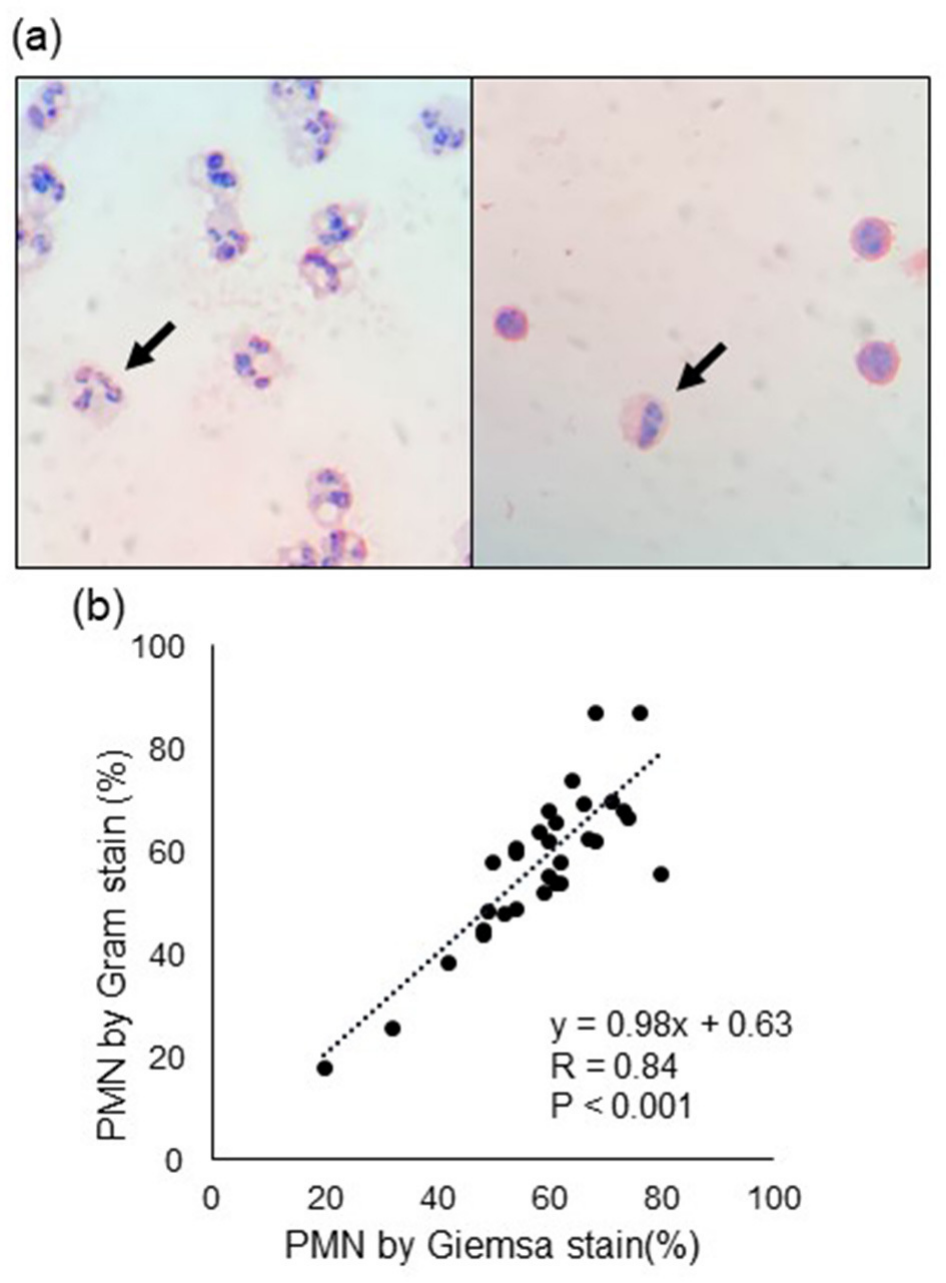
**(A)** Representative images of polymorphonuclear leukocyte (PMN, arrow). The left and right images show high and low PMN counts, respectively. **(B)** Correlation of PMN counts between Gram and Giemsa staining. R denotes the correlation coefficient.

Representative images of PMN and mononuclear cells with Gram staining are shown in Figure 2A, and the scatter plot and correlation between Giemsa and Gram staining are depicted in Figure 1B. The regression line calculated from least-squares analysis between PMN counts from Giemsa (x) and Gram (y) strains was y = 0.98x + 0.63. Correlations between SCC, PMN, cytokines, and SAA are shown in Table 3. SCC were significantly positively correlated with PMN (*p* = 0.005), IL-6 (*p* = 0.001), TNF-α (*p* < 0.001), and SAA (*p* < 0.001) concentrations in milk, whereas PMN were significantly negative correlated with milk IL-10 concentration (*p* = 0.006).

**Table 3:** Correlation among somatic cell counts, polymorphonuclear leukocyte counts, and inflammation indicators in milk.

	**SCC**	**PMN**	**IL-6**	**TNF-α**	**IL-8**	**IL-10**	**SAA**
SCC							
PMN	0.315^**^						
IL-6	0.338^**^	−0.068					
TNF-α	0.400^***^	−0.008	0.782^***^				
IL-8	−0.129	−0.054	0.135	0.178			
IL-10	−0.0003	−0.274^**^	0.204	0.140	−0.158		
SAA	0.681^***^	0.062	0.372^***^	0.306^**^	−0.23^***^	0.015	

PMN, polymorphonuclear leukocyte; IL, interleukin; TNF, tumor necrosis factor; SAA, serum amyloid A; SCC, somatic cell counts.

^**^ and ^***^ represent 0.01, and 0.001, respectively.

## 4 Discussion

Gram staining is often used in clinical settings to identify causal pathogens prior to the availability of culture results (21); however, it has not been used in the diagnosis of mastitis in dairy cows. One reason for this is the difficulty in detecting pathogens in microscopic images of stained milk smears owing to their complex and non-homogeneous background. Therefore, we previously suggested that a smear of concentrated milk cells, suspended in saline through centrifugation, was useful for detecting mastitis-causing pathogens (4). Given that unnecessary antimicrobial use in food-producing animals, such as dairy cows, should be reduced as antimicrobial resistance in dairy animals may threaten public health (22), a rapid method to detect the causal pathogens of mastitis should be developed. This study was conducted to investigate the potential for rapid diagnosis of mastitis, including causal pathogens and inflammation status.

In this study, we demonstrated the sensitivity of each type of *Staphylococcus, Streptococcus, Enterobacteriales*, and NF-GNB. In the clinical setting, the sensitivity and specificity of sputum Gram staining have been discussed for each bacterial species. For example, a systematic review and Bayesian meta-analysis showed that the sensitivity and specificity of sputum Gram stain to diagnose the causal pathogens of community-acquired pneumonia was 0.69 and 0.91 for *Streptococcus pneumoniae*, and 0.76 and 0.97 for *Haemophilus influenzae*, respectively (12). To measure the specificity of Gram staining, it is necessary to identify the bacterial species based on their stained color and morphology under a microscope because this situation means bacterial culture is negative and species cannot be identified from the colonies on agar. For example, in sputum Gram staining, Gram-positive diplococci and Gram-negative coccobacilli were identified as *S. pneumoniae* and *H. influenzae*, respectively. As the type of causal pathogen of mastitis varies and it is difficult to distinguish bacterial species only by the morphology of bacterial cells, this study did not measure the specificity for each type of bacteria.

In this study, the sensitivity and specificity of the MGS was 0.67 and 0.90 for Gram-positive, and 0.50 and 1.00 for Gram-negative bacteria, respectively. We previously demonstrated the MGS method using centrifugation, which was conducted in another clinical center by another laboratory technician. In our previous study, the sensitivity and specificity were 0.84 and 0.86 for Gram-positive, and 0.50 and 0.95 for Gram-negative bacteria, respectively (4). The sensitivity of Gram-positive bacteria in this study was relatively low compared to that in the previous study. Although both studies used the same protocol, differences in interpretation may have influenced their results (21). In a clinical setting, the sensitivity and specificity of urine Gram stain to detect bacteriuria in urinary tract infections were 97.3% and 73.8%, respectively (23). To detect bacterial meningitis, the sensitivity of cerebrospinal fluid Gram staining was approximately 50 to 90% in adults (24). Compared to Gram staining for community-acquired pneumonia, urinary tract infection, and meningitis, the accuracy of MGS is comparable to that reported in clinical settings. However, the reproducibility of these results warrants further investigation.

The sensitivity of Gram-positive bacteria was reportedly higher than that of Gram-negative bacteria. As the background of microscopic images shows low contrast, Gram-negative bacteria are more difficult to detect than Gram-positive bacteria (25). The previous research found that the sensitivity of sputum Gram staining for *H. influenzae, Moraxella catarrhalis, K. pneumoniae*, and *P. aeruginosa* was 60.9, 68.2, 39.25, and 22.2, respectively (26). In this study, the sensitivity of the MGS for Enterobacteriaceae and NF-GNB was 0.18 and 0.71, respectively. Thus, the morphology of Enterobacteriaceae, including *E. coli* and *K. pneumoniae*, may be difficult to distinguish using a microscopic background. Another possible reason is that the bacterial load in milk may affect the sensitivity of Gram staining; thus, this study quantified the pathogens.

The detection limit of the MGS calculated from the ROC curve cut-off was 1,560 cfu/mL. The sensitivity for pathogens with more than 1,560 cfu/mL was 0.85; with < 1,560 cfu/mL was 0.45; and with 10,000 cfu/mL or more was 0.95. Among Gram-negative bacteria, bacterial loads of Enterobacteriaceae and NF-GNB were not significantly different (Figure 2); however, only one strain (9.1%, 1/11) of Enterobacteriaceae and four strains of NF-GNB (26.7%, 4/15) showed >10^4^ cfu/mL. This may explain the low sensitivity of Enterobacteriaceae. In Gram-positive bacteria, the mean and median cfu values of *Staphylococcus* species were higher than those of *Streptococcus* species (Table 2), but their bacterial loads were not significantly different (Figure 2). Moreover, eight strains (24.2%, 8/33) of *Staphylococcus* species and six strains (33.3%, 6/18) of *Streptococcus* species showed >10^4^ cfu/mL. Thus, a low bacterial load (< 10^4^ cfu/mL) may increase the difficulty in detecting pathogens. When compared with another rapid detection method, Nagasawa et al. suggested that the antibody-coated immune-chromatographic strip test for detecting *S. aureus* in mastitic milk has a high sensitivity (100%) when bacterial load exceeds 10^4^ cfu/mL (27). The general detection limit for Gram staining has been reported to be 10^4^-10^5^ cfu/mL (28) whereas MGS showed a sensitivity of 10^3^-10^4^ cfu/mL. In the current method, milk cells were concentrated 10 times with centrifugation, which may explain the low detection limit of the MGS. Therefore, MGS may have a sufficient detection limit for mastitis-causing pathogens prior to culturing.

For the PMN counts, the results were similar for both Gram staining and Giemsa staining. Therefore, it was possible to accurately count the PMN by counting the Gram-stained milk cells. In this study, SCC showed a significantly positive correlation with the concentrations of proinflammatory cytokines and markers (IL-6, TNF-α, and SAA), whereas PMN showed a significantly negative correlation with anti-inflammatory cytokine (IL-10) concentrations. These results may explain the utility of SCC as an indicator of inflammation. Johnzon et al. observed similar changes in SCC; in experimental lipopolysaccharide-induced mastitic milk, the concentrations of IL-6 and TNF-α increased simultaneously at 2 h, with a peak at 24 h, and decreased at 120 h after lipopolysaccharide infusion (29). Therefore, although there is variation in the time since the onset of inflammation in the spontaneous mastitis cases in this study, the correlations among SCC, IL-6, and TNF-α in milk were confirmed because these dynamics are similar during inflammation. In contrast, PMN counts did not correlate with these proinflammatory cytokines and markers but were negatively correlated with IL-10 concentrations. IL-10 is mainly produced by lymphocytes, mast cells, eosinophils, macrophages, and dendritic cells (30). As milk with a low PMN count has a high proportion of lymphocytes and macrophages among somatic cells (18), the negative correlation between PMN count and IL-10 concentration in milk may be explained by the proportion of IL-10-producing cells. Therefore, PMN counts may be a useful tool for determining changes in the inflammation of mammary glands. To better understand milk PMN counts, it is necessary to experimentally elucidate the relationship between the changes in each cytokine and PMN count during the inflammatory stage.

This study is the first to demonstrate the accuracy of the MGS, including the detection limits for different types of mastitis-causing bacteria. Considering the sensitivity, specificity, and detection limit suggested in this study, MGS could help clinical veterinarians develop treatment strategies at the initial examination. Further, this may facilitate the selection of appropriate antibiotics in veterinary clinics, where the use of highly controlled medical devices is limited. This study is the first to suggest that milk PMN counts are significantly and negatively correlated with IL-10 concentration in mastitic milk. However, it remains unclear whether treatment decisions based on the MGS affect mastitis prognosis and antimicrobial use in veterinary settings. Therefore, clinical trials on MGS should be conducted and a larger sample size is needed to reach to any conclusion in the future studies.

## Data Availability

The raw data supporting the conclusions of this article will be made available by the authors, without undue reservation.
